# Supplementary material for: Trends and between-Physician Variation in Laboratory Testing: A Retrospective Longitudinal Study in General Practice
Source: J Clin Med. 2020 Jun 8;9(6):1787. doi: 10.3390/jcm9061787 (PMC7355885; doi:10.3390/jcm9061787)
Supplement: Supplementary file 1 [file jcm-09-01787-s001.pdf]

Supplementary Material

Table S1. Results of mixed-effect logistic regression analysis for complete blood count.

		**Full model**		
Consultations, *n*	1,406,441			
**Fixed effects**	**β (SE)**	**OR (95% CI)**	**Wald's χ^2**	***p*-Value**
Intercept	-1.93 (0.03)	0.145 (0.138–0.152)	-76	<0.001
Male sex	-0.111 (0.004)	0.894 (0.888–0.901)	-29	<0.001
Age (10 years)	-0.014 (0.001)	0.986 (0.984–0.988)	-16	<0.001
Time (10 years)	0.042 (0.007)	1.04 (1.03–1.06)	6.0	<0.001
**Random effects**	**Variance estimate**	**Group members, *n***		
Patient ID	0.69	567,067		
GP ID	0.25	389		
		**Null model**		
**Fixed effects**	**β (SE)**	**OR (95% CI)**	**Wald's χ^2**	***p*-Value**
Intercept	-1.99 (0.02)	0.137 (0.130–0.144)	-81	<0.001
Time (10 years)	0.029 (0.002)	1.030 (1.026–1.034)	4.2	<0.001
**Random effects**	**Variance estimate**	**ICC**		
Patient ID	0.69			
GP ID	0.25	0.059		

Abbreviations: β, coefficient estimate; SE, standard error; OR, odds ratio; CI, confidence interval

Table S2. Results of mixed-effect logistic regression analysis for C-reactive protein.

		**Full model**		
Consultations, *n*	1,191,421			
**Fixed effects**	**β (SE)**	**OR (95% CI)**	**Wald's χ^2**	***p*-Value**
Intercept	-2.59 (0.05)	0.075 (0.067–0.083)	-49	<0.001
Male sex	-0.164 (0.005)	0.85 (0.84–0.86)	-34	<0.001
Age (10 years)	-0.066 (0.001)	0.936 (0.934–0.938)	-58	<0.001
Time (10 years)	0.44 (0.01)	1.55 (1.52–1.58)	42	<0.001
**Random effects**	**Variance estimate**	**Group members, *n***		
Patient ID	0.97	526,366		
GP ID	1.06	375		
		**Null model**		
**Fixed effects**	**β (SE)**	**OR (95% CI)**	**Wald's χ^2**	***p*-Value**
Intercept	-2.69 (0.05)	0.068 (0.061–0.074)	-55	<0.001
Time (10 years)	0.393 (0.002)	1.48 (1.47–1.49)	38	<0.001
**Random effects**	**Variance estimate**	**ICC**		
Patient ID	1.02			
GP ID	1.09	0.202		

Abbreviations: β, coefficient estimate; SE, standard error; OR, odds ratio; CI, confidence interval¨

Table S3. Results of mixed-effect logistic regression analysis for electrolytes (sodium, chloride, potassium).

**Full model**				
Consultations, *n*	470,727			
**Fixed effects**	**β (SE)**	**OR (95% CI)**	**Wald's χ^2**	***p*-Value**
Intercept	-3.74 (0.04)	0.024 (0.022–0.026)	-97	<0.001
Male sex	0.117 (0.009)	1.12 (1.10–1.15)	13	<0.001
Age (10 years)	0.264 (0.002)	1.303 (1.297–1.308)	111	<0.001
Time (10 years)	0.29 (0.03)	1.34 (1.25–1.43)	8.4	<0.001
**Random effects**	**Variance estimate**	**Group members, *n***		
Patient ID	0.90	258,070		
GP ID	0.30	239		
**Null model**				
**Fixed effects**	**β (SE)**	**OR (95% CI)**	**Wald's χ^2**	***p*-Value**
Intercept	-3.54 (0.04)	0.029 (0.027–0.031)	-98	<0.001
Time (10 years)	0.506 (0.005)	1.66 (1.64–1.68)	15	<0.001
**Random effects**	**Variance estimate**	**ICC**		
Patient ID	1.04			
GP ID	0.30	0.065		

Abbreviations: β, coefficient estimate; SE, standard error; OR, odds ratio; CI, confidence interval

Table S4. Results of mixed-effect logistic regression analysis for erythrocyte sedimentation rate.

**Full model**				
Consultations, *n*	1,249,853			
**Fixed effects**	**β (SE)**	**OR (95% CI)**	**Wald's χ^2**	***p*-Value**
Intercept	-5.02 (0.05)	0.006 (0.006–0.007)	-108	<0.001
Male sex	-0.013 (0.009)	0.99 (0.97–1.00)	-1.5	<0.001
Age (10 years)	0.143 (0.002)	1.153 (1.149–1.159)	67	<0.001
Time (10 years)	-0.46 (0.02)	0.63 (0.61–0.65)	-26	0.13
**Random effects**	**Variance estimate**	**Group members, *n***		
Patient ID	1.35	523,747		
GP ID	0.77	372		
**Null model**				
**Fixed effects**	**β (SE)**	**OR (95% CI)**	**Wald's χ^2**	***p*-Value**
Intercept	-4.92 (0.04)	0.007 (0.007–0.008)	-111	<0.001
Time (10 years)	-0.349 (0.004)	0.705 (0.699–0.711)	-22	<0.001
**Random effects**	**Variance estimate**	**ICC**		
Patient ID	1.34			
GP ID	0.76	0.141		

Abbreviations: β, coefficient estimate; SE, standard error; OR, odds ratio; CI, confidence interval

Table S5. Results of mixed-effect logistic regression analysis for fasting glucose.

**Full model**				
Consultations, *n*	1,237,762			
**Fixed effects**	**β (SE)**	**OR (95% CI)**	**Wald's χ^2**	***p*-Value**
Intercept	-4.06 (0.06)	0.017 (0.015–0.019)	-67	<0.001
Male sex	0.219 (0.006)	1.24 (1.23–1.26)	39	<0.001
Age (10 years)	0.181 (0.001)	1.198 (1.195–1.201)	128	<0.001
Time (10 years)	0.01 (0.01)	1.01(0.99–1.04)	1.1	0.26
**Random effects**	**Variance estimate**	**Group members, *n***		
Patient ID	0.76	526,735		
GP ID	1.27	354		
**Null model**				
**Fixed effects**	**β (SE)**	**OR (95% CI)**	**Wald's χ^2**	***p*-Value**
Intercept	-3.85 (0.05)	0.021 (0.019–0.024)	-70	<0.001
Time (10 years)	0.150 (0.003)	1.16 (1.15–1.17)	13	<0.001
**Random effects**	**Variance estimate**	**ICC**		
Patient ID	0.82			
GP ID	1.23	0.231		

Abbreviations: β, coefficient estimate; SE, standard error; OR, odds ratio; CI, confidence interval

Table S6. Results of mixed-effect logistic regression analysis for ferritin.

**Full model**				
Consultations, *n*	697,805			
**Fixed effects**	**β (SE)**	**OR (95% CI)**	**Wald's χ^2**	***p*-Value**
Intercept	-3.56 (0.03)	0.028 (0.027–0.030)	-136	<0.001
Male sex	-0.90 (0.01)	0.405 (0.396–0.413)	-84	<0.001
Age (10 years)	-0.091 (0.002)	0.913 (0.909–0.917)	-39	<0.001
Time (10 years)	0.38 (0.04)	1.47 (1.37–1.57)	11	<0.001
**Random effects**	**Variance estimate**	**Group members, *n***		
Patient ID	1.29	353,110		
GP ID	0.14	302		
**Null model**				
**Fixed effects**	**β (SE)**	**OR (95% CI)**	**Wald's χ^2**	***p*-Value**
Intercept	-4.43 (0.02)	0.012 (0.011–0.012)	-229	<0.001
Time (10 years)	0.309 (0.007)	1.36 (1.34–1.38)	8.7	<0.001
**Random effects**	**Variance estimate**	**ICC**		
Patient ID	1.52			
GP ID	0.09	0.018		

Abbreviations: β, coefficient estimate; SE, standard error; OR, odds ratio; CI, confidence interval

Table S7. Results of mixed-effect logistic regression analysis for glycated hemoglobin.

Full model				
Consultations, *n*	1,393,351			
**Fixed effects**	**β (SE)**	**OR (95% CI)**	**Wald's χ^2**	***p*-Value**
Intercept	-4.52 (0.04)	0.011 (0.010–0.012)	-112	<0.001
Male sex	0.276 (0.008)	1.32 (1.30–1.34)	36	<0.001
Age (10 years)	0.252 (0.002)	1.287 (1.282–1.292)	130	<0.001
Time (10 years)	0.63 (0.01)	1.87 (1.82–1.92)	45	<0.001
**Random effects**	**Variance estimate**	**Group members, *n***		
Patient ID	1.65	558,358		
GP ID	0.61	384		
**Null model**				
**Fixed effects**	**β (SE)**	**OR (95% CI)**	**Wald's χ^2**	***p*-Value**
Intercept	-4.24 (0.04)	0.014 (0.013–0.015)	-113	<0.001
Time (10 years)	0.833 (0.03)	2.30 (2.29–2.32)	65	<0.001
**Random effects**	**Variance estimate**	**ICC**		
Patient ID	1.71			
GP ID	0.56	0.101		

Abbreviations: β, coefficient estimate; SE, standard error; OR, odds ratio; CI, confidence interval

Table S8. Results of mixed-effect logistic regression analysis for lipid profile (high-density lipoprotein, low-density lipoprotein, total cholesterol, triglycerides).

Full model				
Consultations, *n*	957,350			
**Fixed effects**	**β (SE)**	**OR (95% CI)**	**Wald's χ^2**	***p*-Value**
Intercept	-4.49 (0.03)	0.011 (0.011–0.012)	-163	<0.001
Male sex	0.476 (0.008)	1.61 (1.58–1.64)	57	<0.001
Age (10 years)	0.224 (0.002)	1.251 (1.245–1.256)	102	<0.001
Time (10 years)	-0.23 (0.02)	0.80 (0.77–0.83)	-11	<0.001
**Random effects**	**Variance estimate**	**Group members, *n***		
Patient ID	1.43	446,055		
GP ID	0.22	333		
**Null model**				
**Fixed effects**	**β (SE)**	**OR (95% CI)**	**Wald's χ^2**	***p*-Value**
Intercept	-4.094 (0.000)	0.017 (0.016–0.018)	-157	<0.001
Time (10 years)	-0.048 (0.000)	0.95 (0.94–0.96)	-2.9	0.004
**Random effects**	**Variance estimate**	**ICC**		
Patient ID	1.44			
GP ID	0.22	0.044		

Abbreviations: β, coefficient estimate; SE, standard error; OR, odds ratio; CI, confidence interval

Table S9. Results of mixed-effect logistic regression analysis for liver enzymes (alanine transaminase, aspartate transaminase, gamma-glutamyl transferase, alkaline phosphatase).

**Full model**				
Consultations, *n*	777,721			
**Fixed effects**	***β* (SE)**	**OR (95% CI)**	**Wald's χ^2**	***p*-Value**
Intercept	-3.35 (0.02)	0.035 (0.034–0.037)	-135	<0.001
Male sex	0.117 (0.006)	1.12 (1.11–1.14)	18	<0.001
Age (10 years)	0.102 (0.002)	1.107 (1.104–1.111)	65	<0.001
Time (10 years)	0.10 (0.02)	1.10 (1.06–1.15)	5.1	<0.001
**Random effects**	**Variance estimate**	**Group members, *n***		
Patient ID	0.90	379,302		
GP ID	0.18	304		
**Null model**				
**Fixed effects**	***β* (SE)**	**OR (95% CI)**	**Wald's χ^2**	***p*-Value**
Intercept	-3.24 (0.02)	0.039 (0.038–0.041)	-133	<0.001
Time (10 years)	0.192 (0.004)	1.21 (1.20–1.22)	9.8	<0.001
**Random effects**	**Variance estimate**	**ICC**		
Patient ID	0.88			
GP ID	0.17	0.040		

Abbreviations: *β*, coefficient estimate; SE, standard error; OR, odds ratio; CI, confidence interval

Table S10. Results of mixed-effect logistic regression analysis for prothrombin time/international normalized rate.

**Full model**				
Consultations, *n*	846,437			
**Fixed effects**	***β* (SE)**	**OR (95% CI)**	**Wald's χ^2**	***p*-Value**
Intercept	-9.79 (0.04)	0.000 (0.000–0.000)	-262	<0.001
Male sex	0.38 (0.03)	1.46 (1.37–1.55)	12	<0.001
Age (10 years)	0.400 (0.008)	1.49 (1.47–1.52)	47	<0.001
Time (10 years)	-1.10 (0.03)	0.33 (0.31–0.35)	-37	<0.001
**Random effects**	**Variance estimate**	**Group members, *n***		
Patient ID	33.5	387,459		
GP ID	0.0	291		
**Null model**				
**Fixed effects**	***β* (SE)**	**OR (95% CI)**	**Wald's χ^2**	***p*-Value**
Intercept	-9.71 (0.03)	0.000 (0.000–0.000)	-341	<0.001
Time (10 years)	-0.688 (0.006)	0.503 (0.497–0.509)	-24	<0.001
**Random effects**	**Variance estimate**	**ICC**		
Patient ID	42.6			
GP ID	0.0	0.000		

Abbreviations: *β*, coefficient estimate; SE, standard error; OR, odds ratio; CI, confidence interval

Table S11. Results of mixed-effect logistic regression analysis for serum creatinine.

Full model				
Consultations, *n*	1,336,100			
**Fixed effects**	**β (SE)**	**OR (95% CI)**	**Wald's χ^2**	***p*-Value**
Intercept	-3.15 (0.03)	0.043 (0.041–0.045)	-124	<0.001
Male sex	0.140 (0.004)	1.15 (1.14–1.16)	33	<0.001
Age (10 years)	0.180 (0.001)	1.197 (1.195–1.199)	173	<0.001
Time (10 years)	0.27 (0.01)	1.31 (1.29–1.34)	28	<0.001
**Random effects**	**Variance estimate**	**Group members, *n***		
Patient ID	0.39	556,819		
GP ID	0.24	381		
**Null model**				
**Fixed effects**	**β (SE)**	**OR (95% CI)**	**Wald's χ^2**	***p*-Value**
Intercept	-2.97 (0.03)	0.051 (0.049–0.054)	-118	<0.001
Time (10 years)	1.049 (0.003)	2.85 (2.84–2.87)	41	<0.001
**Random effects**	**Variance estimate**	**ICC**		
Patient ID	0.47			
GP ID	0.24	0.059		

Abbreviations: β, coefficient estimate; SE, standard error; OR, odds ratio; CI, confidence interval

Table S12. Results of mixed-effect logistic regression analysis for thyroid-stimulating hormone.

Full model				
Consultations, *n*	756,935			
**Fixed effects**	**β (SE)**	**OR (95% CI)**	**Wald's χ^2**	***p*-Value**
Intercept	-3.81 (0.03)	0.022 (0.021–0.023)	-129	<0.001
Male sex	-0587 (0.008)	0.556 (0.547–0.565)	-71	<0.001
Age (10 years)	0.009 (0.002)	1.009 (1.006–1.013)	4.9	<0.001
Time (10 years)	0.04 (0.03)	1.04 (0.99–1.10)	1.6	0.11
**Random effects**	**Variance estimate**	**Group members, *n***		
Patient ID	0.84	365,022		
GP ID	0.24	290		
**Null model**				
**Fixed effects**	**β (SE)**	**OR (95% CI)**	**Wald's χ^2**	***p*-Value**
Intercept	-4.05 (0.03)	0.017 (0.016–0.018)	-140	<0.001
Time (10 years)	0.046 (0.006)	1.05 (1.04–1.06)	1.7	0.09
**Random effects**	**Variance estimate**	**ICC**		
Patient ID	0.89			
GP ID	0.24	0.054		

Abbreviations: β, coefficient estimate; SE, standard error; OR, odds ratio; CI, confidence interval

Table S13. Results of mixed-effect logistic regression analysis for urinalysis.

Full model				
Consultations, *n*	863,457			
**Fixed effects**	***β* (SE)**	**OR (95% CI)**	**Wald's χ^2**	***p*-Value**
Intercept	-4.45 (0.05)	0.012 (0.011–0.013)	-88	<0.001
Male sex	-0.17 (0.01)	0.84 (0.83–0.86)	-18	<0.001
Age (10 years)	0.057 (0.002)	1.058 (1.053–1.063)	24	<0.001
Time (10 years)	-0.33 (0.03)	0.72 (0.68–0.76)	-12	<0.001
**Random effects**	**Variance estimate**	**Group members, *n***		
Patient ID	1.87	433,802		
GP ID	0.81	326		
**Null model**				
**Fixed effects**	***β* (SE)**	**OR (95% CI)**	**Wald's χ^2**	***p*-Value**
Intercept	-4.48 (0.05)	0.011 (0.010–0.013)	-90	<0.001
Time (10 years)	-0.278 (0.005)	0.757 (0.750–0.764)	-11	<0.001
**Random effects**	**Variance estimate**	**ICC**		
Patient ID	1.83			
GP ID	0.82	0.137		

Abbreviations: *β*, coefficient estimate; SE, standard error; OR, odds ratio; CI, confidence interval

Table S14. Results of mixed-effect logistic regression analysis for vitamin B12.

Full model				
Consultations, *n*	655,448			
**Fixed effects**	***β* (SE)**	**OR (95% CI)**	**Wald's χ^2**	***p*-Value**
Intercept	-4.74 (0.06)	0.009 (0.008–0.010)	-81	<0.001
Male sex	-0.48 (0.01)	0.619 (0.605–0.632)	-44	<0.001
Age (10 years)	0.029 (0.003)	1.029 (1.024–1.034)	11	<0.001
Time (10 years)	0.31 (0.04)	1.36 (1.26–1.48)	7.5	<0.001
**Random effects**	**Variance estimate**	**Group members, *n***		
Patient ID	0.97	324,820		
GP ID	0.85	257		
**Null model**				
**Fixed effects**	***β* (SE)**	**OR (95% CI)**	**Wald's χ^2**	***p*-Value**
Intercept	-4.91 (0.06)	0.007 (0.007–0.008)	-85	<0.001
Time (10 years)	0.323 (0.008)	1.38 (1.36–1.40)	7.8	<0.001
**Random effects**	**Variance estimate**	**ICC**		
Patient ID	0.98			
GP ID	0.85	0.166		

Abbreviations: *β*, coefficient estimate; SE, standard error; OR, odds ratio; CI, confidence interval

Table S15. Results of mixed-effect logistic regression analysis for vitamin D.

Full model				
Consultations, *n*	652,162			
**Fixed effects**	**β (SE)**	**OR (95% CI)**	**Wald's χ^2**	***p*-Value**
Intercept	-4.88 (0.04)	0.008 (0.007–0.008)	-127	<0.001
Male sex	-0.62 (0.01)	0.54 (0.52–0.55)	-47	<0.001
Age (10 years)	0.035 (0.003)	1.036 (1.029–1.042)	12	<0.001
Time (10 years)	0.63 (0.05)	1.88 (1.71–2.06)	13	<0.001
**Random effects**	**Variance estimate**	**Group members, *n***		
Patient ID	1.23	330,650		
GP ID	0.36	275		
**Null model**				
**Fixed effects**	**β (SE)**	**OR (95% CI)**	**Wald's χ^2**	***p*-Value**
Intercept	-5.08 (0.04)	0.006 (0.006–0.007)	-136	<0.001
Time (10 years)	0.646 (0.007)	1.91 (1.88–1.94)	15	<0.001
**Random effects**	**Variance estimate**	**ICC**		
Patient ID	1.23			
GP ID	0.37	0.075		

Abbreviations: β, coefficient estimate; SE, standard error; OR, odds ratio; CI, confidence interval

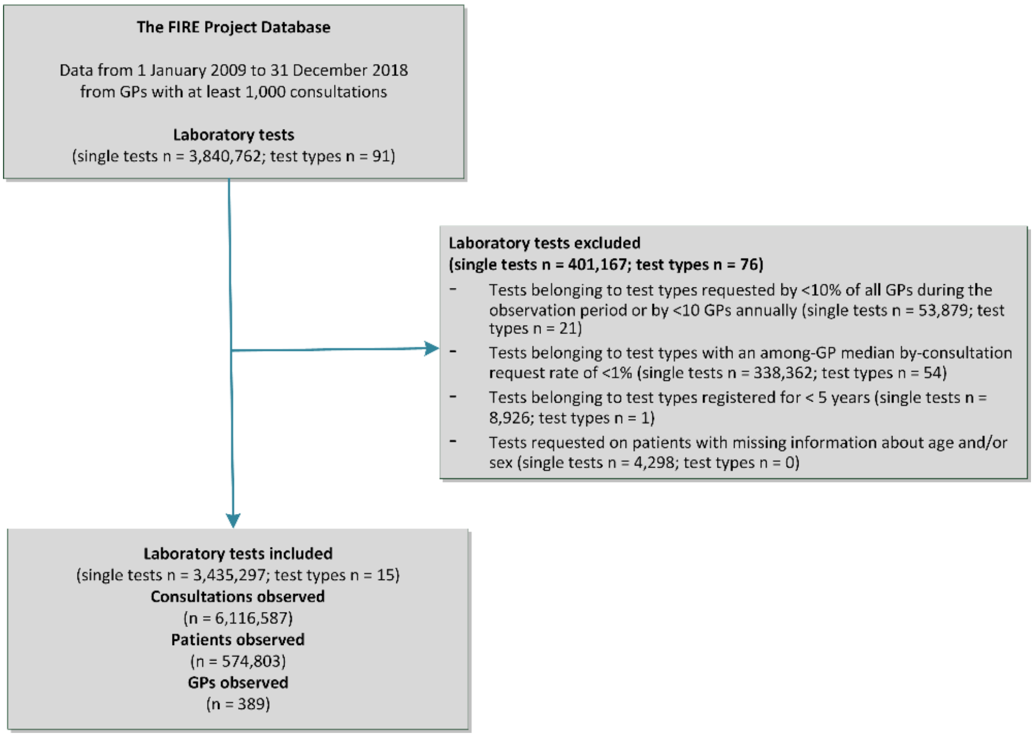

Figure S1. Data selection process. Abbreviations: GP, general practitioner.

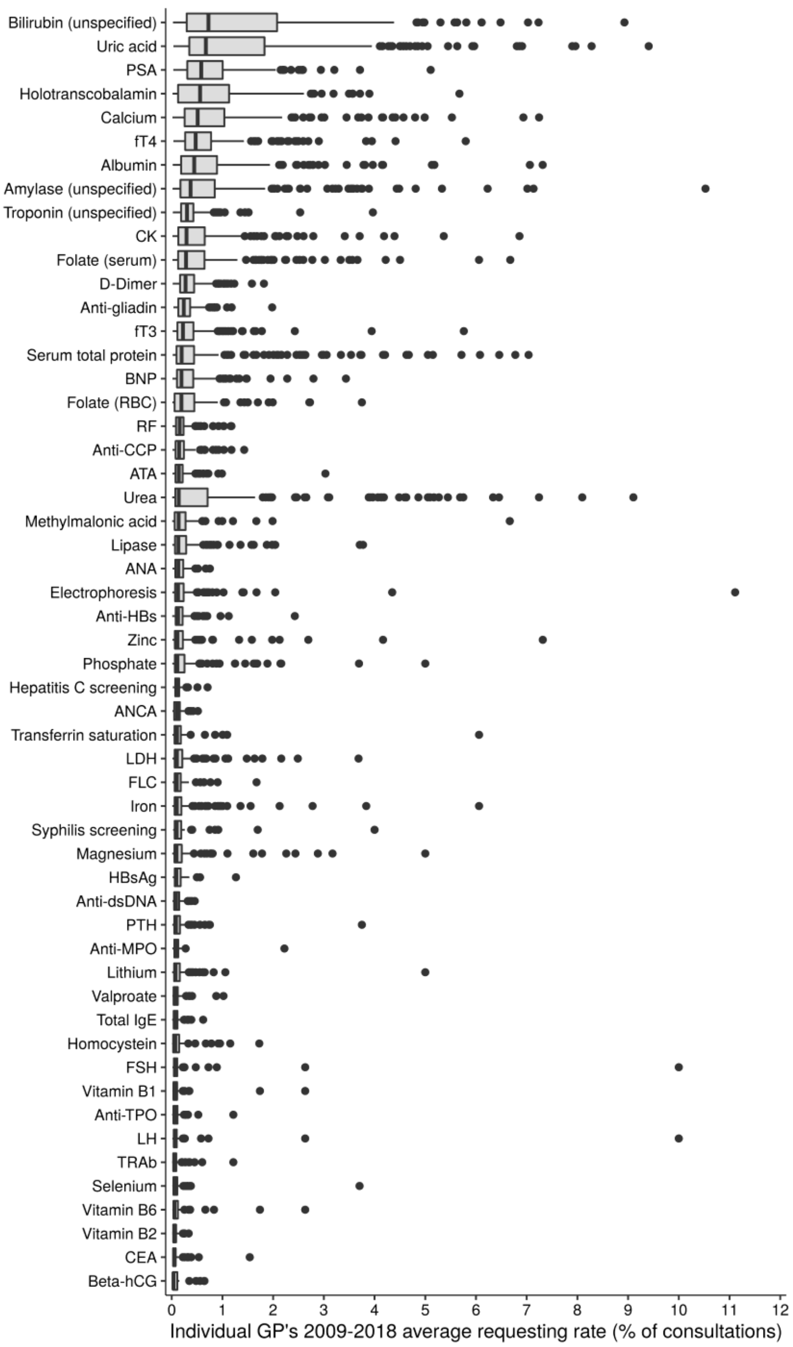

Figure S2. Crude among-general practitioner (GP) distributions of 2009–2018 average specific laboratory testing rates. Test types excluded from analysis for occurring with an among-GP median frequency of <1% of consultations (before aggregation to test panels). Type-specific laboratory testing rates were calculated for each GP as the percentage of consultations during the GP's observation period involving a request of the respective test type. Abbreviations: ANA, antinuclear antibodies; ANCA, anti-neutrophil cytoplasmic antibodies; anti-CCP, anti-cyclic citrullinated peptide; anti-dsDNA, anti-double stranded DNA; anti-HBs, anti-hepatitis B surface antigen; anti-MPO, anti-myeloperoxidase; anti-TPO, anti-thyroid peroxidase; ATA, anti-transglutaminase antibodies; Beta-HCG, Beta-human chorionic gonadotropin; BNP, B-type natriuretic peptide; CEA, carcinoembryonic antigen; CK, creatine kinase; FLC, free light chains; FSH, follicle stimulating hormone; fT3, free triiodothyronine; fT4, free thyroxine; HbsAg, hepatitis B surface antigen; LDH, lactate dehydrogenase; LH, luteinizing hormone; PSA, prostate-specific antigen; PTH, parathyroid hormone; RBC, red blood cells; RF, rheumatoid factor; TRAb, thyrotropin receptor antibodies.
